# Autonomic Modulation and Body Composition in Educators: Physiological Associations in the Workplace Context

**DOI:** 10.1002/brb3.71473

**Published:** 2026-06-19

**Authors:** Estela Álvarez‐Gallardo, Andrea Calderón García, Pilar González‐Sanz, Pedro Belinchón‐deMiguel, Vicente Javier Clemente‐Suárez

**Affiliations:** ^1^ Department of Nursing, Faculty of Medicine, Health and Sports Universidad Europea de Madrid Villaviciosa de Odón Spain; ^2^ Department of Pharmacy and Nutrition, Faculty of Biomedical Sciences Universidad Europea de Madrid Villaviciosa de Odón Spain; ^3^ Research Group in Culture, Education and Society University of the Coast Barranquilla Colombia

**Keywords:** adipose tissue, autonomic modulation, body composition, educators, heart rate variability, skeletal muscle

## Abstract

**Purpose:**

This study examined the relationship between body composition and cardiac autonomic nervous system activity in educators. The objective was to explore the associations between body fat mass (BFM) and fat‐free mass (FFM) with different heart rate variability (HRV) indices in a sample of educators and to assess whether FFM moderates the relationship between BFM and autonomic function.

**Method:**

A descriptive cross‐sectional study was conducted with 253 educators from compulsory and higher education institutions during the 2022–2023 academic year. Autonomic regulation was assessed via HRV, and body composition was measured using bioelectrical impedance analysis.

**Findings:**

Individuals with higher fat mass exhibited less favorable autonomic modulation, evidenced by lower values of the root mean square of successive differences (RMSSD), a time‐domain index that reflects parasympathetic activity and the recovery capacity of the autonomic nervous system. Those with greater fat mass also showed a higher mean heart rate, whereas participants with higher FFM displayed more efficient cardiac activity, with lower mean, minimum, and maximum heart rates. In addition, FFM partially moderated the relationship between BFM and mean heart rate. By contrast, a higher total body water content was associated with a shift toward sympathetic dominance, reflected in higher low‐frequency (LF) components—which represent a mixture of sympathetic and parasympathetic activity—and lower high‐frequency (HF) components, which predominantly reflect parasympathetic activity. Conversely, individuals with higher FFM presented lower mean, minimum, and maximum heart rates, suggesting more efficient autonomic cardiac regulation.

**Conclusion:**

Overall, the findings demonstrate that body composition—particularly FFM and BFM—significantly influences HRV and autonomic nervous system modulation in educators, underscoring the importance of considering body composition in the assessment of autonomic function and in the design of personalized health strategies.

## Abstract 2

Body composition is significantly associated with autonomic nervous system modulation in educators. Higher body fat mass is related to reduced heart rate variability and lower parasympathetic activity, whereas greater fat‐free mass is associated with more efficient autonomic regulation and lower heart rate. These findings highlight the relevance of body composition in autonomic function assessment and its potential role in the development of personalized health strategies.

## 1 Introduction

Educators are exposed to multiple occupational risk factors, such as cognitive demands, academic pressure, irregular schedules, and chronic stress (Francisco et al. 2021), which can negatively affect autonomic balance and trigger physiological and psychosocial alterations (Shanafelt et al. 2022). Autonomic nervous system (ANS) dysfunctions are associated with metabolic disorders and visceral fat accumulation (Köchli et al. 2020; Gruber et al. 2025), with obesity indicators being potential markers of autonomic imbalance (Rastović et al. 2017).

In this context, heart rate variability (HRV) is recognized as a useful, sensitive, and non‐invasive tool for studying cardiac autonomic modulation. It is accepted as a key indicator of cardiovascular health, emotional stability, and neurophysiological balance (Shanafelt et al., 2022; Shaffer and Ginsberg, 2017). HRV is defined as the fluctuations in time between consecutive heartbeats. This variability can be analyzed using time‐domain indicators (such as the Root Mean Square of Successive Differences [RMSSD] and the percentage of intervals differing by more than 50 ms [pNN50]), frequency‐domain analysis (such as power in low‐frequency [LF] and high‐frequency [HF] bands and their ratio [LF/HF]), as well as nonlinear methods. These metrics allow for the assessment of ANS influence on heart rate (Lehrer and Gevirtz, 2014).

Moreover, recent research indicates that non‐invasive interventions targeting the central nervous system, such as brain stimulation, can modulate autonomic activity and alter HRV parameters, thereby reinforcing the physiological validity of these indices as non‐invasive measures of autonomic modulation (Messina et al. 2024).

The ANS includes the sympathetic nervous system (SNS), activated in stressful situations, and the parasympathetic nervous system (PNS), predominant during relaxation states (Lehrer and Eddie, 2013). Higher HRV is associated with emotional resilience, while reduced HRV indicates autonomic dysfunction (Lehrer et al. 2000).

Moreover, individuals experiencing high levels of daily stress tend to show lower R–R interval values, as well as reductions in RMSSD and PNN50—both indicators of R–R interval variability and autonomic recovery capacity. These individuals also tend to exhibit a higher LF/HF ratio, suggesting decreased parasympathetic activity. LF power is associated with SNS activity, while HF power reflects PNS activity and vagal tone. Therefore, the LF/HF ratio provides a measure of the balance between sympathetic and parasympathetic activity, serving as a key indicator of the body's response to risk factors (Lehrer and Eddie, 2013).

Although physiological modulation is increasingly documented and interest in the field is growing, few studies have explored how specific components of body composition, such as body fat mass (BFM) and fat‐free mass (FFM), relate to cardiac autonomic regulation in academic populations. An increase in BFM has been linked to higher sympathetic activity and lower vagal modulation, forming a dysfunctional autonomic profile prone to inflammation (Oliveira et al. 2020). In contrast, higher FFM—indicative of adequate muscle reserves—is associated with better vagal response and a more favorable autonomic profile, suggesting a protective effect against physiological stress (Cvijetić et al. 2023).

These associations have also been observed in other pathophysiological contexts (Du Plessis et al. 2015), such as in breast cancer survivors, where significant differences in HRV and body composition have been identified compared to women without an oncological history (Vis et al. 2024). Additionally, normalized visceral fat rating (VFR) has been correlated with various HRV indices, reinforcing the importance of considering these structural parameters in the evaluation of autonomic modulation.

These findings suggest that structural aspects of the human body, beyond their anthropometric value, may play a key role in the regulation of stress physiology. Understanding this interaction could provide valuable insights for designing prevention and health promotion strategies targeted at educators—a population often underestimated in physiological research. Physical health directly influences the functioning of the autonomic nervous system (ANS), affecting both academic performance and quality of life (Mendes et al. 2024).

Furthermore, multivariate analyses such as *K*‐means clustering and principal component analysis (PCA) were incorporated to identify distinct physiological profiles and latent structures within the HRV data. This study examines the relationship between body composition (BFM and FFM) and HRV in educators, evaluating whether FFM moderates the impact of BFM on autonomic function. It is hypothesized that higher BFM is associated with reduced vagal modulation and increased heart rate, whereas higher FFM may act as a protective factor against physiological stress.

## 2 Materials and Methods

### 2.1 Design, Context, and Participants

To ensure the methodological validity of the study, the recommended guidelines for high‐quality cross‐sectional research were followed (Von Elm et al. 2007).

A descriptive cross‐sectional study was conducted with the participation of 253 educators, of whom 27.30% (*n* = 69) were men and 72.70% (*n* = 184) were women. Participants were recruited from both compulsory and higher education institutions. Of the total sample, 79.20% (*n* = 200) taught in educational centers within the Community of Madrid—covering compulsory education and non‐university higher education—whereas 20.80% (*n* = 53) worked at Spanish universities (tertiary education).

Inclusion criteria required participants to be actively employed as educators in the selected institutions during the 2022–2023 academic year. Exclusion criteria included having a diagnosed cardiovascular disease, chronic psychiatric disorders, or any condition that could interfere with HRV measurements, such as the use of beta‐blockers or other medications that alter heart rate.

Participants were voluntarily recruited through invitations sent via institutional mailing lists and announcements made during meetings with educators. Detailed information about the study objectives, procedures, and ethical considerations was provided prior to obtaining informed consent. To participate, educators were required to sign informed consent forms and agree to undergo HRV measurements. Participation was entirely voluntary, and individuals could withdraw at any time without negative consequences.

The sample size was determined through a power analysis. A medium effect size (*d* = 0.5) was used for the calculation, which allowed for the detection of moderate differences between groups. A statistical power of 80% was established, ensuring an 80% probability of detecting a true effect, with a significance level of 0.05, setting a 5% threshold for accepting a Type I error. Based on these parameters, the minimum required sample size was estimated to be 200 participants. However, to reduce the impact of potential dropouts and improve the study's validity, a larger sample was recruited.

Data collection was scheduled across two time windows during the 2022–2023 academic year (beginning and end) to facilitate multicenter logistics and coordination among participating institutions. All centers followed the same academic calendar, which allowed for the homogenization of measurement timelines. It is important to note that the beginning of the academic year may be influenced by emotional and mental health factors related to the so‐called ‘post‐vacation effect,’ characterized by increased stress and adaptation difficulties following the summer break. This phenomenon may affect baseline HRV levels during the early days of the academic term (Sonnentag et al. 2010). Additionally, stress levels were expected to rise as the academic year progressed due to increasing mental fatigue and workload demands (Castilla‐Gutiérrez et al. 2021).

From a methodological perspective, several relevant covariates were considered for the analysis. In particular, sex differences in HRV and body composition are well documented in the scientific literature (Koenig and Thayer 2016) and were therefore included as control factors. Likewise, the educational level of the teaching staff (university vs. non‐university) was taken into account, as it could influence stress levels and, consequently, the autonomic parameters evaluated.

After data collection, all information was anonymized, and any identifying details of the participants were removed. The study was approved by the Institutional Review Board (CIPI/213006.55), and informed consent was obtained from all participants in accordance with the bioethical principles established in the latest version of the Declaration of Helsinki (WMA 2013).

Although participants with chronic conditions that could affect HRV were excluded, validated psychological questionnaires were administered to assess the emotional status of the educators, including symptoms of anxiety, depression, and stress. However, the results of these instruments were not incorporated into the present analysis and therefore do not form part of the study outcomes. As a result, the conclusions are based exclusively on physiological parameters. This methodological decision was taken to focus the analysis on physiological indicators, although it is acknowledged that the inclusion of these psychological data in future publications would enable a more comprehensive characterization of the emotional impact on autonomic function.

### 2.2 Instruments

Autonomic modulation was assessed through heart rate variability (HRV) analysis using the Polar V800 monitor (Polar, Kempele, Finland), a device validated in previous studies (Belinchón‐Demiguel and Clemente‑Suárez, 2018; Belinchón‐de Miguel et al., 2024). Each participant completed a single recording session, conducted in a seated position, at complete rest, and breathing spontaneously (without guided respiration). Prior to the measurements, specific instructions were provided to ensure recording reliability for both BIA and HRV. Participants also received general information regarding the importance of maintaining a relaxed state in order to preserve natural physiological conditions and avoid external interference in autonomic responses. The evaluations were conducted under the participants’ usual daily conditions. They were instructed to attend the session after having their habitual breakfast and maintaining normal hydration, without receiving specific instructions regarding tobacco or alcohol consumption or physical activity prior to the assessment.

Each recording lasted 6 min (short‐term measurement, ≥5 min), which provides a reliable estimation of HRV indices. All sessions were conducted between 8:30 a.m. and 12:00 p.m. to minimize the influence of circadian variations. Participants maintained their usual breakfast routine and normal hydration. They were assessed individually in quiet rooms with stable temperature and controlled environmental conditions to ensure a standardized setting. The scheduling was adapted to the availability of the teaching staff and distributed across three consecutive days, which facilitated sample representativeness without disrupting their professional routines.

For the BIA assessment, participants were asked to attend the session without wearing any metallic objects. These instructions were provided verbally, and compliance was verified prior to each measurement.

Data were processed using Kubios HRV Scientific software (version 4.1.0), following the guidelines of the Task Force of the European Society of Cardiology and the North American Society of Pacing and Electrophysiology (Malik et al. 1996). The following parameters were analyzed: minimum (HRmin), mean (HRmean), and maximum heart rate (HRmax); RMSSD; pNN50; LF/HF ratio; low‐frequency (LF) power and normalized high‐frequency (HFn) power; as well as nonlinear indices SD1 and SD2. These indicators have been widely validated as sensitive markers of sympathetic and parasympathetic activity. Specifically, RMSSD and PNN50 reflect vagal modulation, while LF, HF, and their ratio allow for estimation of autonomic balance. SD1 and SD2 provide additional information on the complexity of cardiac control.

Since several of these parameters may be influenced by mean heart rate, adjustments were considered when appropriate, following methodological recommendations by Bailón et al. (2011) and Bolea et al. (2016), who propose correction techniques to improve comparative validity in clinical and experimental contexts (Bailón et al. 2011; Bolea et al. 2016). To ensure data quality, all RR recordings were manually inspected, and artifacts or abnormal beats were corrected using the built‐in Kubios algorithms (threshold‐based detection and automatic interpolation). Only recordings with <5% corrections were included, in accordance with standard signal‐quality criteria.

Body composition was assessed via bioelectrical impedance analysis (BIA) using the InBody 720 device (Biospace Co., Ltd., Seoul, Korea), which is widely recognized for its precision and reproducibility. To standardize hydration status, measurements were performed in mid‐morning, with participants under light solid fasting (having already consumed their usual breakfast), following immediate bladder voiding, with no vigorous physical exercise in the preceding 12 h, and avoiding caffeine/alcohol over the same period. All assessments were conducted barefoot and without metallic objects, and the cleanliness and drying of hands and feet were verified prior to electrode placement. Because total body weight does not discriminate among physiological compartments, this variable was excluded; instead, body fat mass (BFM) and fat‐free mass (FFM) were prioritized due to their relevance to autonomic regulation. Although the BIA device provides multiple parameters—such as body mass index (BMI), percent body fat (PBF), visceral fat rating (VFR), total body water (TBW), intracellular water (ICW), and extracellular water (ECW)—BFM and FFM were retained as the primary predictors in regression models because they represent physiological compartments with clear functional implications for autonomic modulation. To monitor potential hydration variability, TBW, ICW, and ECW ratios reported by the device were systematically reviewed, and the ECW/TBW ratio (edema index) was documented when available, confirming the absence of outliers prior to analysis. This decision is grounded in previous studies demonstrating consistent associations between these indicators and autonomic nervous system activity (Bruce et al. 2025). The device underwent the manufacturer's recommended verification and calibration procedures at the start of each assessment day, and a quality checklist was applied before every measurement.

With respect to validity and reliability, the literature has documented good agreement of the InBody 720 and other multifrequency BIA platforms with reference techniques (e.g., DXA) in adults, as well as acceptable repeatability coefficients under standardized conditions. In our protocol, non‐consecutive duplicates were collected within the same session (same operator) for a small subgroup to confirm reading stability; intra‐day coefficients of variation remained within acceptable ranges (<2%–3% for FFM and <3%–5% for BFM). Nevertheless, variables such as VFR, TBW, ICW, and ECW were considered in exploratory and correlational analyses given their potential value as complementary biomarkers. In particular, VFR has shown relevant correlations with HRV indices in recent research, suggesting its utility for future investigations aimed at characterizing more specific autonomic profiles. All body‐composition‐related acronyms were defined consistently throughout the manuscript to ensure methodological clarity.

### 2.3 Statistical Procedures

All statistical analyses were performed using SPSS version 29.0 (IBM Corp., Armonk, NY, USA) and Python 3.11. A descriptive analysis was conducted to examine the sociodemographic and occupational characteristics of the participants. To assess the normality of the distributions, the Shapiro–Wilk test was applied. Since several variables did not meet the assumptions of normality, non‐parametric tests were primarily used. Group comparisons were conducted using Student's *t*‐test, the Mann–Whitney *U* test, and the chi‐square (*χ* ^2^) test, depending on the nature and distribution of each variable. In cases where more than 20% of the frequencies were below 5, Fisher's exact test was applied to ensure statistical validity.

Within the experimental design, descriptive statistical measures were incorporated, including central tendency (mean), dispersion (standard deviation), and 95% confidence intervals, with the aim of adequately characterizing the sample and ensuring the robustness of the analyses. Relevant independent variables were identified and controlled, including age, which was considered a key confounding variable in this study due to its potential influence on HRV and body composition. Age was included as a covariate in multivariate models to avoid bias in estimating the physiological effects associated with occupational stress. This variable was included as a covariate in the multivariate models in order to adjust the physiological associations under study, without linking these effects to psychological stress levels.

To examine group differences in autonomic function, Mann–Whitney *U* tests were used to compare HRV indices between participants with high and low FFM and between those with high and low BFM, based on median splits. Effect sizes were reported as r values.

Spearman correlation analyses were conducted to evaluate associations between body composition parameters (BFM, FFM, BMI, percent body fat [PBF], TBW, ICW, and ECW) and HRV variables (mean heart rate [Mean HR], RMSSD, pNN50, LF, HF, LF/HF ratio, SD1, and SD2). Correlations with *p*‐values < 0.05 were considered statistically significant.

To analyze the relationship between body composition and heart rate variability (HRV) parameters, multiple linear regression models were employed. These models allowed the prediction of individual HRV variables based on body fat mass (BFM) and fat‐free mass (FFM), adjusting for age and sex as a priori covariates. Body mass index (BMI) and physical activity level were not included as covariates in the main models, given that BMI was incorporated in the exploratory correlational analyses and its potential collinearity with BFM/FFM could distort the estimation of their independent effects, while physical activity data were not homogeneously available for the entire sample. The independent contribution of each predictor was evaluated using standardized regression coefficients (*β*), the adjusted coefficient of determination (*R* ^2^), and the associated *p*‐values. Because some dependent variables did not meet normality assumptions according to the Shapiro–Wilk test, various transformations (logarithmic, square‐root, Box–Cox, and no transformation) were explored to improve the distribution of residuals. The selected transformation was the one that maximized the *p*‐value of the normality test and provided an adequate model fit. Inferential validity was assessed through complementary diagnostic procedures, including visual inspection of residuals, tests for homoscedasticity (Breusch–Pagan), normality (Kolmogorov–Smirnov), and Q–Q plots. In general, only some residuals exhibited approximate normality, constant variance, and independence.

Therefore, due to the nature of some distributions (asymmetry or the presence of extreme values), generalized linear models (GLMs) with a Gamma family and log link function were used in cases where the dependent variable exhibited marked skewness, as was the case for RMSSD. This approach allowed appropriate modeling of continuous and strictly positive variables without requiring residual normality. The GLMs included BFM, FFM, age, and sex as a priori predictors. As in the linear regression models, BMI and physical activity were not incorporated as covariates for the reasons previously described. Model adequacy was evaluated through residual plots and Q–Q plots, which indicated a satisfactory fit. Results are reported as coefficient estimates, standard errors, *p*‐values, and 95% confidence intervals.

In addition, moderation analyses were conducted to determine whether FFM moderated the relationship between BFM and specific HRV outcomes. For these models, an interaction term (BFM × FFM) was included alongside the main effects.

For the principal component analysis (PCA), all HRV variables were standardized (z‐scores). The suitability of the data matrix was verified using the KMO index and Bartlett's test of sphericity. Component retention followed a priori criteria: Kaiser's rule (eigenvalues > 1), visual inspection of the scree plot to identify the inflection point, and an adequate threshold of cumulative explained variance to support substantive interpretation. Orthogonal rotation (varimax) was applied when appropriate to enhance the interpretability of factor loadings.

For the unsupervised classification, *k*‐means clustering was performed using standardized BFM, FFM, and HRV indices. The choice of the optimal number of clusters was based on internal validation indices, including the average silhouette coefficient, the Calinski–Harabasz index (CH), and the Davies–Bouldin index (DBI), selecting the solution that maximized silhouette and CH values and minimized DBI. Cluster stability was assessed through multiple random initializations (n ≥ 100) with a reproducible seed. As a sensitivity analysis, clustering was replicated using the factor scores of the retained principal components, yielding qualitatively consistent results.

Finally, a *k*‐means cluster analysis was applied to identify distinct participant profiles based on BFM, FFM, and HRV variables. The optimal number of clusters was determined using the aforementioned criteria (silhouette, CH, and DBI), and cluster characteristics were reported using descriptive statistics. Emerging profiles were interpreted according to patterns in HRV and body composition.

All statistical tests were two‐tailed, and the significance level was set at *p* < 0.05.

## 3 Results

Table 1 presents a descriptive analysis of the sociodemographic and occupational characteristics of the participants. The mean age was 42.18 years (SD = 8.95), with no significant differences between sexes (*F* = 0.688; *p* = 0.166). The age group distribution (<43 years vs. ≥ 43 years) was balanced, with no significant differences (*χ* ^2^ = 0.831; *p* = 0.386). The average teaching experience was 12.86 years (SD = 9.57).

**TABLE 1:** Characteristics of survey respondents.

		Male	Female	Total	*p*‐value
		66 (28.10%)	169 (71.90%)	235 (100%)	
Age (years)	Mean (SD)	40.94 (8.26)	42.66 (9.18)	42.18 (8.95)	*F* = 0.688 *p* = 0.166
Age group	< *P*50 (43 years)	36 (54.55%)	81 (47.92%)	117 (49.80%)	*χ* ^2^ = 0.831 *p* = 0.386
	≥ *P*50 (43 years)	30 (45.45%)	88 (52.08%)	118 (50.20%)	
Teaching level	Early Childhood and Primary Education	22 (33.30%)	82 (48.52%)	104 (44.20%)	*χ* ^2^ = 8.055 *p* = 0.018*
	Secondary Education/ Baccalaureate/ Vocational Training	31 (46.97%)	47 (27.81%)	78 (33.20%)	
	University Teaching	13 (19.70%)	40 (23.67%)	53 (22.60%)	
Workplace	Concerted center	31 (46.97%)	77 (45.56%)	108 (46.00%)	*χ* ^2^ = 0.038 *p* = 0.846
	Private center	35 (53.03 %)	92 (54.44%)	127 (54.00%)	
Teaching experience (years)	Mean (SD)	10.77 (8.13)	13.66 (9.98)	12.86 (9.57)	*F* = 4,61 *p* = 0.076

*Significant differences *p* < 0.05. Chi‐squared test for the comparison of prevalences. Mean comparisons by Student's *t*‐test or Mann–Whitney *U* test depending on whether they follow a normal distribution. 1Fisher's exact test applied because more than 20% of the frequencies have a sample size <5.

Abbreviation: SD = standard deviation.

Table 2 shows that participants classified with higher FFM exhibited significantly lower mean heart rate (*U* = 1209, *p* = 0.011, *r* = 0.230), lower minimum heart rate (*U* = 1237, *p* = 0.014, *r* = 0.216), and lower maximum heart rate (*U* = 1258, *p* = 0.020, *r* = 0.205) compared to those with lower FFM. These results suggest a physiological pattern consistent with more efficient autonomic modulation in individuals with higher lean mass. No significant differences were observed in RMSSD, pNN50, SD1, SD2, or in HRV frequency‐domain parameters such as LF, HF, or the LF/HF ratio (Table 1: Comparison of HRV variables between low‐ and high‐FFM groups).

**TABLE 2:** Comparison of HRV variables between low and high FFM groups.

Variable	Low FFM (Mean ± SD)	High FFM (Mean ± SD)	*U*	*p*‐value	Effect size (*r*)
Mean HR (Heart Rate)	78.8 ± 13.9	72.5 ± 13.1	29,775	0.001	0.229
Minimun HR (bpm)	64.3 ± 11.5	60.5 ± 10.1	28,744	0.001	0.191
Maximum HR (bpm)	126.7 ± 95.4	108.5 ± 106.8	29,228	0.001	0.209
RMSSD (ms)	65.2 ± 51.7	61.8 ± 48.0	24,120	0.660	0.021
pNN50 (%)	21.6 ± 20.4	22.3 ± 19.7	22,832	0.586	−0.026
Low frequency (LF) (ms^2^)	62.6 ± 17.2	65.9 ± 17.3	20,910	0.044	−0.097
High frequency (HF) (ms^2^)	37.3 ± 17.1	34.0 ± 17.3	26,198	0.042	0.097
RatioLF.HF	2.6 ± 2.4	3.1 ± 2.9	21,004	0.052	−0.093
SD1 (ms)	46.1 ± 36.6	43.7 ± 33.8	24,083	0.680	0.020
SD2 (ms)	82.2 ± 56.9	78.8 ± 49.4	23,380	0.900	−0.006

*Note*: Variables were compared using the Mann–Whitney *U* test. Effect sizes are reported as *r* values.

Abbreviations: BFM = body fat mass, HF = high frequency, HR = heart rate, LF = low frequency, LF/HF = low‐to‐high frequency ratio, pNN50 = percentage of successive RR intervals differing by more than 50 ms, RMSSD = root mean square of successive differences, SD1 = standard deviation of short‐term variability, SD2 = standard deviation of long‐term variability.

Conversely, as shown in Table 3, participants with higher body fat mass (BFM) exhibited a significantly higher mean heart rate (*U* = 1322, *p* = 0.018, *r* = 0.217) and lower values of RMSSD (*U* = 1355, *p* = 0.042, *r* = 0.192), pNN50 (*U* = 1301, *p* = 0.025, *r* = 0.204), and SD2 (*U* = 1284, *p* = 0.036, *r* = 0.211) compared with those with lower BFM. These findings reflect a less favorable autonomic modulation profile, characterized by reduced parasympathetic activity and lower HRV complexity among participants with higher adiposity (Table 2: Comparison of HRV between low‐ and high‐BFM groups).

**TABLE 3:** Comparison of HRV between low and high BFM groups.

Variable	Low BFM (Mean ± SD)	High BFM (Mean ± SD)	*U*	*p*‐value	Effect size (*r*)
Mean HR (Heat Rate) (bpm)	74.7 ± 13.8	76.6 ± 13.8	21,850	0.196	−0.062
Minimum HR (bpm)	61.2 ± 11.0	63.7 ± 10.9	19,991	0.007	−0.130
Maximum HR (bpm)	116.8 ± 80.9	118.5 ± 119.3	24,058	0.692	0.019
RMSSD (ms)	68.4 ± 51.7	58.4 ± 47.5	26,062	0.054	0.093
pNN50 (%)	24.4 ± 21.0	19.4 ± 18.7	26,884	0.010	0.123
Low frequency (ms^2^)	66.1 ± 16.6	62.4 ± 17.8	26,304	0.034	0.102
High frequency (ms^2^)	33.8 ± 16.6	37.5 ± 17.8	20,780	0.035	−0.101
LF/HF ratio	3.1 ± 2.8	2.6 ± 2.6	26,410	0.028	0.105
SD1 (ms)	48.3 ± 36.4	41.3 ± 33.7	26,078	0.052	0.093
SD2 (ms)	88.4 ± 56.5	72.4 ± 48.5	28,325	0.001	0.176

*Note*: Variables were compared using the Mann–Whitney *U* test. Effect sizes are reported as *r* values.

Abbreviations: BFM = body fat mass, HF = high frequency, HR = heart rate, LF = low frequency, LF/HF = low‐to‐high frequency ratio, pNN50 = percentage of successive RR intervals differing by more than 50 ms, RMSSD = root mean square of successive differences, SD1 = standard deviation of short‐term variability, SD2 = standard deviation of long‐term variability

In addition, a descriptive analysis of the body composition parameters obtained through bioelectrical impedance analysis (BIA) was performed, and the results are summarized in Table 4. This table provides a comprehensive overview of the physiological characteristics of the educators assessed, including body fat mass (BFM), fat‐free mass (FFM), skeletal muscle mass (SMM), total body water (TBW), intracellular water (ICW), extracellular water (ECW), percent body fat (PBF), and body mass index (BMI). Values are presented as means ± standard deviation or as medians with their corresponding interquartile ranges, depending on the distribution of each variable. This information allows for contextualizing the body composition profiles of the sample and facilitates the interpretation of the findings in terms of autonomic modulation, without linking them to subjective indicators of stress.

**TABLE 4:** Body profile of the Teacher Group according to BIA analysis.

Variable	Group summary
BFM	18.05 [13.22–22.88]
FFM	49.96 ± 10.74
SMM	27.60 ± 6.53
TBW	36.60 ± 7.92
ICW	22.70 ± 5.02
ECW	13.91 ± 2.92
PBF	27.19 ± 9.13
BMI	23.60 [21.60–25.98]

Abbreviations: BFM = body fat mass, BMI = body mass index, ECW = extracellular water, FFM = fat‐free mass, ICW = intracellular water, PBF = percent body fat, SMM = skeletal muscle mass, TBW = total body water.

Although the median was used as the criterion to divide the sample into high and low FFM/BFM groups, this strategy was complemented by a detailed analysis of anthropometric and physiological parameters. Table 4 includes complete descriptive measures that allow for precise characterization of body profiles. Additionally, correlation analyses were conducted between these parameters and HRV indices, reinforcing the validity of the classification used and enabling interpretation of the results based on the individual physiology of the participants.

### 3.1 Significant Correlations Between Body Composition and HRV Indices

Significant correlations were found between body composition parameters and HRV indices. Fat‐free mass (FFM) was negatively correlated with mean HR (Mean HR; *r* = −0.265, *p* < 0.001) and maximum HR (*r* = −0.226, *p* < 0.001), indicating that individuals with greater lean mass exhibited lower basal and peak cardiac activity. Similarly, skeletal muscle mass (SMM) (*r* = −0.258, *p* < 0.001), total body water (TBW) (*r* = −0.264, *p* < 0.001), intracellular water (ICW) (*r* = −0.259, *p* < 0.001), and extracellular water (ECW) (*r* = ‐0.268, *p* < 0.001) were all inversely associated with Mean HR. Body height also showed a significant inverse correlation with minimum HR (*r* = −0.232, *p* < 0.001). Conversely, higher body fat percentage (PBF) was positively correlated with minimum HR (*r* = 0.227, *p* < 0.001), consistent with a less advantageous autonomic modulation profile in individuals with higher adiposity.

### 3.2 Multiple Linear Regression Models

Multiple linear regression analyses were conducted to evaluate the predictive value of BFM and FFM on various HRV parameters. For each main dependent variable, different statistical transformations—logarithmic, square root, Box–Cox, or no transformation—were explored to optimize the normality of residuals. The selected transformation was the one that yielded the highest *p*‐value in the Shapiro–Wilk normality test and demonstrated good model fit.

In the case of mean heart rate (MeanHR), the Box–Cox transformation resulted in an approximately normal distribution of residuals (*p* = 0.0657). The model was statistically significant (*F* = 13.63, *p* = 1.8e–10, adjusted *R* ^2^ = 0.104). BFM was positively and significantly associated with MeanHR (*β* = 0.001, *p* = 0.0207), while FFM showed a non‐significant inverse association (*β* = –0.001, *p* = 0.146). Age had a significant negative effect (*β* = –0.002, *p* = 0.000453), and sex was also a significant predictor (*β* = 0.040, *p* = 0.0304).

For the LF/HF ratio (RatioLF_HF), the Box–Cox transformation also produced a normal distribution of residuals (*p* = 0.717). The model was significant (*F* = 8.76, *p* = 8.4e–07, adjusted R^2^ = 0.067). BFM showed a non‐significant inverse association (*β* = –0.004, p = 0.392), while FFM was inversely and significantly associated (*β* = –0.014, *p* = 0.0309). Age did not reach statistical significance (*β* = 0.009, *p* = 0.0502), and sex was a significant predictor (*β* = –0.702, *p* = 1.33e–05).

In most models, the Box–Cox transformation improved residual normality, although in some cases this assumption was not fully met. BFM showed inverse and significant associations with parasympathetic activity indices such as RMSSD, pNN50, SD1, and SD2. FFM was significantly associated with reduced LF power and a lower LF/HF ratio. Additionally, both age and sex demonstrated significant effects in several models.

These findings suggest that higher BFM is consistently associated with reduced parasympathetic activity, while FFM independently contributes to lower heart rate and alterations in autonomic tone, as reflected in LF and HF components.

Overall, the results refer to associations adjusted for age and sex; the absence of additional adjustment for BMI and physical activity should be taken into account when interpreting the magnitude of these associations.

### 3.3 Modelos Lineales Generalizados (GLM)

Since the assumption of normality was not fully met, GLM with a gamma family and logarithmic link function was employed. GLM models were fitted for each of the main HRV indices (MeanHR, HRmin, HRmax, RMSSD, pNN50, LF, HF, SD1, and SD2), using BFM, FFM, and age as predictors. The selection of model family and link function was based on the nature of each dependent variable, with the Gamma family used for asymmetric and positive variables, the Gaussian family for normally distributed variables, and the binomial family for proportions.

For MeanHR, the significant predictors were BFM, with a positive coefficient (0.170; *p* = 0.041), and age, with a negative coefficient (–0.263; *p* < 0.001), indicating that a higher percentage of body fat is associated with higher mean heart rate, while age is inversely associated. For minimum heart rate (HRmin), BFM was the only significant predictor (coefficient = 0.175; p = 0.009), suggesting that higher fat content predicts a higher minimum heart rate. No significant predictors were identified in the model for maximum heart rate (HRmax).

Regarding parasympathetic variability, both BFM (coefficient = –0.011; *p* = 0.019) and age (coefficient = –0.012; *p* = 0.008) were significant negative predictors for RMSSD, indicating that both higher fat content and older age are associated with lower parasympathetic activity. For pNN50, age was the only significant predictor (coefficient = –0.030; *p* = 0.036), showing that parasympathetic variability decreases with age.

In the frequency domain, LF power was significantly influenced by FFM (coefficient = –0.005; *p* = 0.035) and age (coefficient = 0.003; *p* = 0.042), suggesting that greater lean mass and age affect LF band power.

For SD1 and SD2 indices, BFM (coefficient = –0.011; *p* = 0.020 for SD1; coefficient = –0.011; *p* = 0.006 for SD2) and age (coefficient = –0.012; *p* = 0.007 for SD1; coefficient = –0.009; *p* = 0.012 for SD2) were significant negative predictors, reflecting lower heart rate variability and complexity associated with higher body fat and older age.

Overall, the GLM models show that body fat mass and age are the most consistently associated predictors of reduced heart rate variability, both in the time and frequency domains, highlighting their adverse impact on parasympathetic modulation and HRV complexity. Additionally, fat‐free mass also showed significant associations with specific indices, underscoring the importance of body composition and demographic factors in cardiac autonomic modulation.

### 3.4 Moderation Analysis

To examine whether FFM moderated the association between BFM and autonomic modulation, moderation analyses were conducted using interaction models for selected HRV parameters. The model predicting mean HR was statistically significant (F = 11.57, *p* < 0.001, *R* ^2^ = 0.075). FFM was a significant negative predictor (*β* = −0.614, *p* < 0.001), and the interaction term BFM × FFM also reached significance (*β* = 0.016, *p* = 0.035), suggesting that the impact of BFM on mean HR varied depending on FFM levels. In contrast, no significant interaction effects were found for RMSSD (*β* = −0.010, *p* = 0.719) or pNN50 (*β* = −0.003, *p* = 0.759), indicating that the relationship between BFM and these indices of parasympathetic activity was not moderated by FFM.

### 3.5 Cluster Analysis (*K*‐means)

A *k*‐means cluster analysis was conducted using standardized BFM, FFM, and a broad set of HRV indices (z‐scores). The silhouette method indicated that a two‐cluster solution provided the best fit to the data. This choice was further supported by internal validation indices: the *k* = 2 solution yielded the highest average silhouette coefficient and the highest Calinski–Harabasz index, along with the lowest Davies–Bouldin index when compared with alternative k values. Cluster stability was confirmed through multiple random initializations (*n* ≥ 100) using a reproducible seed.

Cluster 0 (*n* = X) was characterized by moderate BFM (19.95 kg), similar FFM (49.76 kg), higher mean heart rate (74.77 bpm), and lower parasympathetic activity (RMSSD = 38.72 ms; pNN50 = 12.71%). In contrast, Cluster 1 (*n* = X) presented slightly lower BFM (16.86 kg), comparable FFM (49.90 kg), markedly higher RMSSD (133.02 ms), elevated pNN50 (47.84%), and a more balanced autonomic profile (LF/HF ratio = 1.16).

These results suggest that autonomic profiles may vary relatively independently of FFM, with a subgroup exhibiting high vagal modulation despite having similar levels of lean mass.

### 3.6 Principal Component Analysis (PCA)

A PCA was conducted to explore the underlying dimensionality of the HRV parameter set. All HRV variables were standardized (z‐scores), and data adequacy was assessed using the Kaiser–Meyer–Olkin (KMO) index and Bartlett's test of sphericity. Component retention followed a priori criteria: (i) Kaiser's rule (eigenvalues > 1), (ii) visual inspection of the scree plot to identify the inflection point, and (iii) an adequate threshold of cumulative explained variance to ensure substantive interpretability. Varimax rotation was applied, when appropriate, to enhance the interpretability of factor loadings.

The first principal component (PC1) explained the largest proportion of variance and was characterized by high positive loadings on RMSSD (0.41), pNN50 (0.38), SD1 (0.41), and SD2 (0.37), reflecting overall parasympathetic modulation and HRV complexity. The second component (PC2) showed strong positive loadings on mean heart rate (0.69), minimum heart rate (0.56), and maximum heart rate (0.42), representing general cardiac activation. The third component (PC3) loaded positively on LF (0.46) and negatively on HF (−0.46), indicating a frequency‐domain contrast between sympathetic and parasympathetic influences. Together, the retained components met the predefined retention criteria (eigenvalues > 1 supported by the scree plot) and accounted for an adequate proportion of cumulative variance for meaningful interpretation. These results support a multidimensional structure of HRV and justify the use of factor scores in subsequent clustering or regression analyses.

## 4 Discussion

The main objective of this study was to examine the relationship between BFM and lean body mass (LBM) with various HRV indices in a sample of educators. Additionally, the potential moderating effect of LBM on the association between BFM and ANS function was explored.

Our hypothesis proposed that higher levels of LBM would be associated with more efficient cardiac autonomic regulation, characterized by a reduction in heart rate (HR). The findings support this hypothesis, as educators with higher LBM exhibited significantly lower mean, minimum, and maximum heart rates compared to those with lower LBM. These results describe associations consistent with more efficient autonomic modulation and greater parasympathetic predominance in individuals with higher lean mass, which translates into lower resting heart rate and greater HRV.

However, no significant differences were found in time‐domain HRV indices (RMSSD, pNN50, SD1, and SD2) or in frequency‐domain parameters (LF, HF, and LF/HF ratio). This pattern may be explained by the dynamic nature of HRV and by the influence of uncontrolled factors within this sample (e.g., age, physical fitness, and interindividual variability), underscoring the need to interpret the findings cautiously and within the observational framework of the study.

Furthermore, it is plausible that the beneficial effects of higher LBM on autonomic regulation manifest primarily through reductions in heart rate rather than through direct changes in HRV indices, as has also been observed in resistance‐training studies involving older adults (Gambassi et al. 2016).

These phenomena may be explained by the positive impact of lean mass on ANS activity, attributable to several physiological mechanisms. First, the beneficial effect of muscle tissue on autonomic activity may be related to the role of the vagus nerve in HR regulation. This mechanism can be explained by the active role of muscle tissue in enhancing baroreceptor sensitivity, contributing to more effective blood pressure regulation and promoting an adaptive autonomic response to physiological demands (Alatawi, 2023).

Second, a higher proportion of LBM has been associated with reduced levels of systemic inflammation, conditions that favor a more stable autonomic function (Shi et al. 2023). HRV, which reflects vagal nerve activity, has shown an inverse relationship with inflammatory markers, suggesting that higher vagal tone may influence the regulation of the inflammatory response (Alen et al. 2021).

Complementary evidence suggests that modulation of cortical excitability can translate into adjustments in the sympathetic–parasympathetic balance, reinforcing the notion of a functionally relevant central–autonomic integration. Within this framework, signals related to cortical evaluation of demands (e.g., prefrontal/insular circuits) may influence autonomic nuclei in the brainstem and, consequently, peripheral outputs that are reflected in HRV parameters (e.g., RMSSD, pNN50, and the LF/HF ratio). Thus, HRV serves as a quantifiable bridge between central processes and autonomic responses, providing a non‐invasive physiological marker of this integration. This perspective, supported by studies demonstrating HRV changes following non‐invasive neuromodulatory interventions targeting the central nervous system, strengthens the theoretical basis for interpreting our findings within a top‐down model of autonomic modulation (Messina et al. 2024).

Third, greater muscle mass is closely linked to better cardiorespiratory capacity, which translates into greater efficiency of the autonomic nervous system (Alatawi, 2023). In this context, peak inspiratory conductance (peak IC) is a relevant indirect marker of respiratory activity, especially in studies exploring autonomic modulation. Spontaneous breathing directly influences HRV, particularly the HF components, which reflect parasympathetic activity mediated by the vagus nerve.

Several studies have shown that greater cardiorespiratory capacity, associated with a higher IC peak, correlates with enhanced vagal modulation and improved physiological adaptation to exertion. Although this parameter was not directly assessed in our sample, its inclusion in future research would allow for a more precise characterization of the respiratory profile and its impact on autonomic function, particularly in populations exposed to high occupational demands (Slomka et al. 2017). This improvement in aerobic capacity favors parasympathetic tone predominance, optimizing HR regulation and facilitating more efficient adaptation to physiological stress (Alatawi, 2023). Thus, individuals with greater muscle mass tend to exhibit more balanced HR regulation, supported by greater efficiency in autonomic function (Alatawi, 2023).

These findings are consistent with previous research showing a positive association between muscle mass and increased PNS activity. For example, Cvijetić et al. (2023) found that participants with higher LBM showed a positive correlation with greater parasympathetic activity, measured through HRV. Similarly, a study comparing autonomic modulation between swimmers and non‐athletes found that higher LBM was associated with better ANS modulation, reflected in increased HRV (Rossi et al. 2014).

Similarly, Mabe‐Castro et al. (2024) analyzed 81 older adults and found that those with greater lean muscle mass exhibited a more favorable cardiac autonomic response, characterized by higher parasympathetic activity and increased HRV. These findings reinforce the hypothesis that preserving muscle mass during aging may promote more efficient autonomic regulation, thereby contributing to cardiovascular health in advanced age.

Additionally, Gambassi et al. (2016) evaluated the impact of a moderate‐intensity strength training program in older women and demonstrated that the increase in lean mass achieved through exercise was associated with significant improvements in HRV. Similarly, a recent study by Aggarwal et al. (2024) found that a higher proportion of lean mass was associated with more balanced autonomic function, reinforcing the key role of healthy body composition in maintaining autonomic homeostasis.

Once again, this pattern is consistent with previous research conducted in athletic populations, where individuals with greater lean mass have been shown to exhibit more robust vagal modulation and greater physiological recovery capacity, reflected in higher RMSSD values and a more balanced LF/HF ratio (Ortigosa et al. 2018). These findings align with the idea that muscle mass plays an active role in optimizing autonomic tone, not only as a structural component but also as a functional modulator of the autonomic nervous system (ANS). Nonetheless, no significant differences in time‐domain HRV indices were identified in our sample, which may be explained by lower levels of physical training or by sustained occupational demands (as contextual factors), without implying the measurement of psychological stress.

The results of this study are consistent with recent literature, supporting the hypothesis that higher LBM is associated with a more favorable autonomic modulation profile, reflected in lower resting heart rate. This finding aligns with greater vagal control and, consequently, a potentially more advantageous cardiovascular profile. These physiological effects highlight the importance of maintaining an adequate body composition, characterized by an optimal proportion of muscle mass, as a strategy associated with improved autonomic balance and cardiovascular function.

Conversely, the initial hypothesis posited that higher body fat mass (BFM) would be associated with reduced vagal modulation and increased heart rate (HR). The findings of this study indicate that participants with higher BFM exhibited significantly higher mean HR, along with reduced HRV indices such as RMSSD, pNN50, and SD2. This pattern is consistent with diminished parasympathetic activity and lower overall HRV complexity in individuals with greater adiposity, which should be interpreted as a physiological association rather than a proven causal mechanism.

Several factors may account for this relationship. First, increased BFM—particularly visceral fat—has been linked to a persistent inflammatory state (Kolb, 2022). Adipose tissue, functioning as an endocrine organ, releases various pro‐inflammatory cytokines, such as interleukin‐6 (IL‐6) and tumor necrosis factor‐α (TNF‐α), which adversely affect ANS regulation (Popko et al. 2010). These cytokines promote an imbalance between the sympathetic and parasympathetic branches of the ANS, leading to greater sympathetic activation and reduced parasympathetic tone, manifested as decreased HRV (Kolb, 2022).

Second, excess body fat is associated with insulin resistance, a key factor in altering autonomic balance (Russo et al. 2021). Insulin resistance has been linked to reduced HRV, reflecting diminished parasympathetic activity (Svensson et al. 2016). This phenomenon may contribute to explaining how metabolic dysregulation related to excess adiposity influences autonomic modulation and cardiovascular function (Solaro et al. 2021).

Third, alterations in fat distribution—particularly visceral fat—have been implicated in autonomic dysfunction (Bai et al. 2023). Visceral fat contributes directly to the release of free fatty acids and adipokines, which can modify ANS function (Gugliucci, 2022).

Several studies have shown that increases in visceral fat promote sympathetic activity and reduce parasympathetic activity, resulting in decreased HRV (Martinez‐Sanchez et al. 2022).

Finally, obesity—particularly excessive accumulation of body fat—has been associated with structural and functional alterations of the ANS (Pollard et al. 2020). Obesity has been described as being related to reduced parasympathetic activity and a relative increase in sympathetic activity (Oliveira et al. 2020). These changes are typically assessed through HRV measurements, with reductions observed in RMSSD and pNN50, which are indicators of parasympathetic modulation [Yadav et al. 2017]. This autonomic imbalance is associated with increased cardiovascular risk, as it reflects a diminished capacity of the ANS to adequately regulate heart rate (Shah et al. 2019).

These findings are consistent with previous studies showing an association between higher BFM and alterations in cardiac autonomic regulation. Specifically, prior research has reported that individuals with obesity exhibit reductions in RMSSD and SD1, along with higher LF/HF ratios (Rossi et al. 2015). Similarly, central adiposity—measured by waist circumference—has been inversely associated with vagal activity, suggesting that greater abdominal fat accumulation may be linked to reduced parasympathetic modulation (Farah et al. 2013). A similar pattern has been observed for BFM, which correlates negatively with parasympathetic activity (Cvijetić et al. 2023).

In addition, another study found that both underweight and overweight women exhibited significant reductions in HRV parameters compared with women of normal body weight, suggesting a less favorable autonomic profile at both extremes of the weight spectrum (Triggiani et al. 2017).

Last, a cross‐sectional study investigating the relationship between inflammatory markers, body composition (BIA), HRV, and physical activity levels in university students reported that higher body fat—particularly visceral fat—was associated with lower HRV, consistent with impaired autonomic function (Hoffmann et al. 2025).

In line with the existing literature, these findings support the notion that higher BFM is associated with reduced vagal modulation and a less balanced autonomic profile. This result should be interpreted as an associative (rather than mechanistic) finding and underscores the importance of controlling adiposity to preserve adequate autonomic regulation.

The results obtained in this study reveal significant associations between HRV indices and various body composition parameters. The observed relationships provide insight into how body mass distribution may influence cardiovascular autonomic functioning, particularly in the regulation of heart rate and its variability. One of the most notable observations was the negative correlation between lean body mass (LBM) and heart rate parameters, both at rest and at peak levels. Specifically, LBM showed an inverse association with mean and maximum heart rate.

These findings are consistent with the notion that individuals with greater lean mass tend to exhibit lower basal and maximal cardiac activity, aligning with enhanced cardiovascular efficiency and improved physiological adaptability, as greater muscle mass is often associated with higher levels of cardiorespiratory fitness. Similarly, other body composition parameters—such as skeletal muscle mass (SMM), total body water (TBW), intracellular water (ICW), and extracellular water (ECW)—showed negative associations with mean heart rate, reinforcing the idea that larger amounts of lean tissue may contribute to more efficient heart rate regulation.

The inverse relationship between height and minimum heart rate suggests that taller individuals, with greater body volume, may present lower resting HR, consistent with greater cardiovascular capacity and better autonomic reserve. Conversely, a higher percentage of body fat (%BF) was positively associated with minimum HR, indicating that individuals with greater adiposity tend to exhibit less efficient autonomic regulation of heart rate.

This finding aligns with prior research suggesting that obesity and elevated adipose tissue levels are associated with alterations in autonomic function—particularly in sympathetic–parasympathetic balance—which can manifest as reduced vagal tone.

### 4.1 Results From Multiple Regression Models and Generalized Linear Models (GLM)

The results obtained through multiple regression models and GLM provide robust evidence regarding the modulatory role of body composition in ANS activity, assessed via HRV parameters. Specifically, both BFM and FFM were confirmed to exert differential influences on autonomic modulation, with relevant implications for the cardiovascular health of educators.

The multiple regression models showed that BFM was inversely and significantly associated with indices of parasympathetic activity (RMSSD, pNN50, SD1, and SD2), whereas FFM was related to decreases in LF power and in the LF/HF ratio. This suggests that greater lean mass may promote a more balanced autonomic profile, whereas higher adiposity tends to reduce vagal modulation.

Complementarily, the GLM models allowed us to address the lack of residual normality and provided a perspective more closely aligned with the distributional characteristics of the data. In these models, BFM emerged as a positive and significant predictor of both mean heart rate (coefficient = 0.170; *p* = 0.041) and minimum heart rate (coefficient = 0.175; *p* = 0.009), indicating that higher body fat content is associated with greater basal cardiac activation. In addition, BFM and age were negatively associated with RMSSD and SD1/SD2, confirming their adverse impact on parasympathetic modulation and HRV complexity.

Age also emerged as a consistent negative predictor across several models, particularly for RMSSD, pNN50, SD1, and SD2, suggesting a progressive decline in parasympathetic activity with aging. This trend has been consistently observed in statistical models that include age as a predictor, indicating a gradual loss of vagal modulation over time. For example, Jiang et al. (2022) demonstrated that older adults exhibit significant reductions in vagal components of HRV accompanied by increased sympathetic tone, reflecting an autonomic imbalance characteristic of physiological aging. This parallel reinforces the internal coherence of the findings observed in the present study.

Taken together, these results show that BFM and age are the factors most consistently associated with reduced cardiac variability, reflecting less efficient autonomic modulation. Although FFM shows more specific effects, it also contributes to explaining individual differences in ANS activity.

From an applied perspective, these findings highlight the importance of considering body composition as a relevant physiological parameter for understanding autonomic modulation. Identifying physiological profiles associated with structural patterns may help guide interventions aimed at improving cardiovascular health and autonomic function in professionals exposed to high occupational demands without implying the direct measurement of subjective stress.

### 4.2 Moderation Analysis: Protective Effect of FFM

To explore the potential moderating effect of FFM on the association between BFM and ANS function, a moderation analysis was conducted. The hypothesis proposed that FFM could attenuate the adverse effects of BFM on autonomic modulation, acting as a structurally relevant factor in physiological regulation.

The results of the study indicate that FFM moderates the impact of BFM on mean heart rate. The interaction term between BFM and FFM was significant (*β* = 0.016, *p* = 0.035), suggesting that the relationship between BFM and mean heart rate varies depending on the level of FFM. This finding implies that greater muscle mass may partially buffer the effect of adiposity on cardiac activation, promoting a more efficient autonomic profile.

However, no significant interaction effects between BFM and FFM were observed for parasympathetic activity indices such as RMSSD (*β* = –0.010, *p* = 0.719) or pNN50 (*β* = –0.003, *p* = 0.759), indicating that the relationship between BFM and these HRV parameters is not moderated by FFM. These results are notable because they show that although FFM clearly influences mean heart rate, its role in specific parasympathetic components appears more limited.

This phenomenon may be explained by the fact that FFM, largely composed of skeletal muscle tissue, plays a key role in ANS modulation and in maintaining cardiovascular homeostasis. Skeletal muscle functions as an endocrine organ, releasing various myokines (such as interleukin‐6 [IL‐6], irisin, and myostatin) during contraction. These molecules exert systemic anti‐inflammatory effects and contribute to improved insulin sensitivity, thereby supporting metabolic and cardiovascular regulation (Pedersen and Febbraio, 2012; Whitham and Febbraio, 2016). Such actions promote optimization of the metabolic profile and reduce chronic sympathetic activation, facilitating lower heart rate and enhanced vagal tone.

Conversely, individuals with higher adiposity typically exhibit low‐grade chronic inflammation, characterized by increased release of pro‐inflammatory cytokines such as tumor necrosis factor‐alpha (TNF‐α) and interleukin‐6 (IL‐6) from adipose tissue (Hotamisligil, 2006). This inflammatory environment promotes increased sympathetic activity and reduced parasympathetic activity, impairing autonomic modulation and leading to elevated heart rate (Alen et al. 2021). Nevertheless, greater muscle mass may mitigate this dysfunction through the release of myokines with anti‐inflammatory properties, promoting metabolic homeostasis and beneficially modulating ANS activity (Lee and Jun, 2019).

Within this context, a meta‐analysis examined the impact of body weight changes on ANS activity. The studies reviewed showed that weight loss—particularly when accompanied by physical exercise—improves HRV, suggesting an increase in parasympathetic activity. These adjustments in HRV are associated with enhanced autonomic function, implying that higher FFM percentages may buffer the negative effects of BFM on ANS regulation (Kuang et al. 2019).

Similarly, a study identified ANS activity and brain structure as key predictors of stress resilience. Although FFM was not directly evaluated, the findings suggest that optimal autonomic function—potentially influenced by higher FFM—supports greater physiological resilience to stress (Carnevali et al. 2018).

An additional review analyzed the impact of body composition on the autonomic regulation of cardiovascular function. The results indicated that higher proportions of FFM are associated with better autonomic function, whereas greater BFM is linked to autonomic dysfunction. These findings support the idea that increasing FFM may counteract the detrimental effects of BFM on ANS function (Smoljo et al. 2020).

Our findings align with this body of literature and suggest that FFM plays a relevant role in autonomic modulation, particularly in relation to mean heart rate. Moreover, FFM appears to moderate the relationship between BFM and this variable, emphasizing the importance of muscle mass in physiological regulation.

Finally, although the present study focused on BFM and FFM as primary predictors, preliminary results suggest that additional bioimpedance‐derived parameters, such as visceral fat ratio (VFR), may provide further insight into autonomic modulation. Incorporating these indicators into future multivariate models could enhance physiological characterization within a strictly biological framework.

### 4.3 Análisis de Conglomerados (*K*‐means): Perfiles Autonómicos

The inclusion of complementary analyses such as *K*‐means clustering and principal component analysis (PCA) was justified by the need to identify latent patterns within the physiological data that could not be captured through univariate analyses. These methods allow for the exploration of the internal structure of heart rate variability (HRV) and body composition data, facilitating the identification of differentiated autonomic profiles. This multivariate approach provides a richer and more functional perspective on autonomic modulation, particularly in occupational contexts with high demands, without implying the evaluation of psychological variables.

The results of the present study provide significant evidence of heterogeneity in cardiac autonomic modulation among individuals with similar levels of lean body mass. Cluster analysis using the *K*‐means method revealed the existence of two clearly differentiated profiles. Specifically, Cluster 1, despite presenting lean mass levels comparable to Cluster 0, exhibited substantially greater parasympathetic modulation, reflected in elevated RMSSD and pNN50 values, along with a lower mean heart rate.

These findings suggest that although lean mass is relevant, it is not the sole determinant of autonomic regulation. Other factors—possibly related to body composition, metabolic status, or cardiorespiratory fitness—may significantly influence autonomic nervous system activity.

### 4.4 Principal Component Analysis (PCA): Latent Structure of HRV

In line with these results, PCA identified underlying dimensions within the HRV parameters. The first principal component grouped variables associated with vagal modulation and HRV complexity, while the second component primarily reflected general cardiac activation. The third component described the balance between sympathetic and parasympathetic influences, highlighting the multidimensional nature of cardiac autonomic control.

This dimensional structure supports the importance of analyzing multiple HRV indicators to achieve a more comprehensive understanding of autonomic regulation, rather than relying on isolated metrics.

The combination of both analytical approaches (K‐means and PCA) reinforces the hypothesis that, beyond traditional anthropometric measures, there are functional patterns of autonomic regulation that may be critical for identifying cardiovascular risk profiles.

Moreover, these findings raise the possibility that interventions aimed at enhancing parasympathetic modulation could be beneficial, even in individuals with seemingly healthy body composition characteristics.

### 4.5 Limitations and Directions for Future Research

This study presents several methodological and contextual limitations that should be considered. First, although validated psychological questionnaires were administered to assess emotional symptoms, their results were not included in the analysis; therefore, the interpretation is based exclusively on physiological parameters. Integrating these data, together with endocrine biomarkers such as cortisol, could provide a more comprehensive understanding of chronic occupational stress (Iob and Steptoe 2019).

The sample size and the geographic concentration in educational institutions in Madrid limit the generalizability of the findings. Future research should expand the sample and diversify educational contexts to improve external validity. Additionally, although the multivariate models were adjusted for age and sex (a priori adjustment), other potentially confounding covariates—such as body mass index (BMI) and physical activity level—were not systematically incorporated, which may have introduced residual confounding. New studies should simultaneously integrate BMI and standardized measures of physical activity (e.g., accelerometry or validated questionnaires) to more accurately estimate the independent effects of body composition on autonomic modulation.

The high proportion of women in the sample (72.7%) and the lack of control for sex‐specific physiological variables—such as menstrual cycle phase and hormonal contraceptive use—may have influenced parasympathetic HRV modulation (Pereira et al., 2023).

Body composition assessment using bioelectrical impedance analysis (BIA), although practical, is less precise than techniques such as dual‐energy X‐ray absorptiometry (DXA), which may have affected measurement reliability (Achamrah et al. 2018). Similarly, additional cardiorespiratory parameters, such as inspiratory capacity peak or maximal oxygen uptake (VO_2_ max), were not collected, although their inclusion would enhance physiological characterization (Abdalla et al. 2025). Finally, assessments were conducted under the participants’ usual daily conditions (routine breakfast and normal hydration), without providing specific instructions regarding tobacco or alcohol consumption or prior physical activity. While this decision increases the ecological validity of the protocol, it also introduces uncontrolled variability in HRV compared to more standardized environments.

Psychosocial factors such as workload, social support, or coping strategies were not evaluated, despite their potential influence on heart rate variability. The absence of longitudinal follow‐up also limits the ability to examine the impact of stress across different stages of professional life and its association with chronic diseases. Moreover, the use of more advanced statistical approaches (e.g., structural equation modeling or machine learning) could improve the predictive capacity of the models. Finally, the use of more precise monitoring devices, such as the Polar H10, is recommended to enhance technological integration in HRV measurement.

### 4.6 Practical Applications

The findings of this study have relevant implications for occupational health, particularly within the educational sector, by informing physiologically oriented strategies to support autonomic function. The evidence regarding the relationship between HRV, autonomic dysfunction, and changes in body composition—particularly increases in body fat percentage—highlights the need for targeted interventions to promote cardiovascular health in this population.

These results underscore the importance of considering both lean mass and fat mass in the design of strategies aimed at optimizing autonomic modulation and preventing cardiovascular diseases associated with ANS dysfunction. Improving body composition—through increasing muscle mass and reducing fat mass—emerges as an effective pathway to enhance autonomic health and reduce the risk of metabolic and cardiovascular disorders.

In this regard, educational institutions, both at the school and university levels, could implement personalized wellness programs focused on promoting regular physical activity, sleep hygiene, progressive aerobic and resistance training tailored to baseline fitness, nutritional counseling, and access to preventive cardiovascular screening. Mind–body practices (e.g., breathing techniques, relaxation protocols, or mindfulness) may also serve as adjuncts to support autonomic balance, alongside access to psychological support when needed (without implying that psychological stress was evaluated in this study). Program design should prioritize objective physiological endpoints (e.g., resting heart rate, HRV indices, blood pressure, and anthropometric markers) in accordance with the scope of the present study.

By prioritizing educators’ cardiometabolic and autonomic health through evidence‐informed, physiology‐focused programs, institutions may contribute to improved well‐being and functional recovery, potentially enhancing participation, satisfaction, and productivity—while acknowledging that the present findings are limited to physiological associations and do not constitute direct measurements of psychological or work‐related stress.

## 5 Conclusions

This study confirms that body composition—specifically fat‐free mass (FFM) and body fat mass (BFM)—is significantly associated with HRV and ANS modulation in educators. Higher BFM was associated with lower HRV and with patterns consistent with greater sympathetic influence, which aligns with a less favorable autonomic profile and a potentially increased cardiovascular risk. Moreover, FFM appeared to partially attenuate the association between higher BFM and increased mean heart rate, acting as a physiologically protective factor in individuals with greater adiposity. These findings reinforce the importance of maintaining an adequate proportion of muscle mass to optimize autonomic regulation and preserve cardiovascular health.

## Author Contributions

**Estela Álvarez‐gallardo**: methodology, software, formal analysis, investigation, data curation, writing – original draft, writing – review and editing. **Andrea Calderón García**: methodology, software, formal analysis, investigation, data curation, resources, supervision, writing – original draft. **Pilar González‐sanz**: writing – review and editing, writing – original draft. **Pedro Belinchón‐demiguel**: conceptualization, investigation, funding acquisition, writing – original draft, writing – review and editing, visualization, validation, methodology, software, formal analysis, project administration, resources, supervision, data curation. **Vicente Javier Clemente–suárez**: conceptualization, investigation, funding acquisition, writing – original draft, methodology, validation, visualization, writing – review and editing, software, formal analysis, project administration, data curation, supervision, resources. All authors have read and agreed to the published version of the manuscript.

## Funding

The authors have nothing to report.

## Institutional Review Board Statement

The study was approved by the Institutional Review Board (CIPI/22.178), and informed consent was obtained from all participants in accordance with the bioethical principles established in the latest version of the Declaration of Helsinki.

## Consent

Informed consent was obtained from all subjects involved in the study. Written informed consent has been obtained from the patient(s) to publish this paper.

## Conflicts of Interest

The authors declare no conflicts of interest.

## Data Availability

The data that support the findings of this study are available from the corresponding author upon reasonable request.
